# Novel technical developments in mass spectrometry imaging in 2020: A mini review

**DOI:** 10.1002/ansa.202000176

**Published:** 2021-03-16

**Authors:** Brooke A. Dilmetz, Yea‐Rin Lee, Mark R. Condina, Matthew Briggs, Clifford Young, Christopher T. Desire, Manuela Klingler‐Hoffmann, Peter Hoffmann

**Affiliations:** ^1^ Future Industries Institute University of South Australia Adelaide Australia; ^2^ Clinical and Health Sciences, Health and Biomedical Innovation University of South Australia Adelaide Australia; ^3^ Discipline of Orthopaedics and Trauma, Adelaide Medical School The University of Adelaide Adelaide Australia

**Keywords:** formalin‐fixed paraffin‐embedded, MALDI, mass spectrometry imaging, reproducibility, workflows

## Abstract

The applicability of mass spectrometry imaging (MSI) has exponentially increased with the improvement of sample preparation, instrumentation (spatial resolution) and data analysis. The number of MSI publications listed in PubMed continues to grow with 378 published articles in 2020‐2021. Initially, MSI was just sensitive enough to identify molecular features correlating with distinct tissue regions, similar to the resolution achieved by visual inspection after standard immunohistochemical staining. Although the spatial resolution was limited compared with other imaging modalities, the molecular intensity mapping added a new exciting capability. Over the past decade, significant improvements in every step of the workflow and most importantly in instrumentation were made, which now enables the molecular analysis at a cellular and even subcellular level. Here, we summarize the latest developments in MSI, with a focus on the latest approaches for tissue‐based imaging described in 2020.

**Abbreviations**
AP‐MALDI: atmospheric pressure matrix‐assisted laser desorption/ionization
AuNPs: gold nanoparticles
BNDM: 1,1′‐binaphthyl‐2,2′‐diamine
CHCA: α‐cyano‐4‐hydroxycinnamic acid
CID: collision‐induce dissociation
CMC: carboxymethyl cellulose
DAN: 1,5‐diaminonaphthalene
DESI: desorption electrospray ionization
DHB: 2,5‐dihydroxybenzoic acid
ECD: electron‐capture dissociation
EFPE: ethanol‐fixed paraffin‐embedded
FF: fresh‐frozen
FFPE: formalin‐fixed paraffin‐embedded
f‐LAESI: fiber‐based laser ablation electrospray ionization
HPMA: poly N‐(hydroxypropyl)‐methacrlyamide
IHC: immunohistochemical
IMS^n^
: ion mobility fragmentation
ITO: indium–tin oxide coated
LA‐ICP‐MS: laser ablation‐inductively coupled plasma‐mass spectrometry
LC: liquid‐chromatography
MALDI: matrix‐assisted laser desorption/ionisation
MIMS: multi‐isotope imaging mass spectrometry
ML: machine learning
MS: mass spectrometry
MSI: mass spectrometry imaging
NN: neural network
OCT: optimal cutting medium
PDMS: polydimethylsiloxane
PLL: poly‐l‐lysine
PTM: posttranslational modifications
PVP: polyvinylpyrrolidone
QC: quality control
ROI: region of interest
SA: sinapinic acid
SIMS: secondary ion mass spectrometry
SOM: self‐organizing map
TEC: tissue extinction coefficient
TIC: total ion count
TIMS: trapped ion mobility mass spectrometer
TOF: time‐of‐Flight

## 1 INTRODUCTION

**FIGURE 1:**
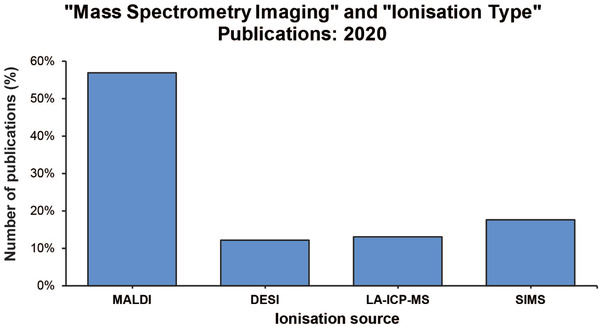
The number of publications related to mass spectrometry imaging from 2020. The search string used in the “search” field was “mass spectrometry imaging” and “MALDI” or “desorption electrospray ionisation NOT laser ablation” or “laser ablation” or “secondary ion”. Data was retrieved from PubMed on 02/12/2020

Mass spectrometry imaging (MSI) was first described over 50 years ago and utilized secondary ion mass spectrometry (SIMS).^1^ Further developments by Hillenkamp and Karas in the 1970s, and their use of a laser microprobe mass analyzer enabled the analysis of small molecules in semiconductors.^2^ However, it was the pioneering work by Caprioli^3^ and Spengler^4^ that demonstrated the use of matrix‐assisted laser‐desorption/ionization mass spectrometry (MALDI‐MSI) for the analysis of proteins and peptides in a biological context that led to the widespread use of MSI in clinical applications. MSI is the acquisition of distinct mass spectra within a virtual equidistant grid across a tissue of choice. The individual mass spectra can then be displayed as intensity maps for each *m/z* feature. This technique retains the spatial information of the tissue analyzed in contrast to other mass spectrometry (MS) approaches, which require homogenization prior to analysis. This technology has been used extensively for determining the spatial distribution of peptides,^5^ lipids,^6^ glycans,^7^ and metabolites^8^ for plant,^9^ food,^10^ and pharmaceutical^11^ applications. The development and implementation of quality control (QC) parameters are imperative to minimize the inherent issues associated with various MSI technologies (such as ion suppression and sample heterogeneity). Therefore, improvements to the MSI pipeline (sample preparation, equipment, and data analysis) are required. At the forefront of MSI technologies and the most widely used is MALDI‐MSI (Figure 1). Here, we will provide a brief overview of the latest developments of MSI with respect to sample preparation, instrumentation, and data analysis, with an emphasis on MALDI‐MSI in 2020.

## 2 SAMPLE PREPARATION

**FIGURE 2:**
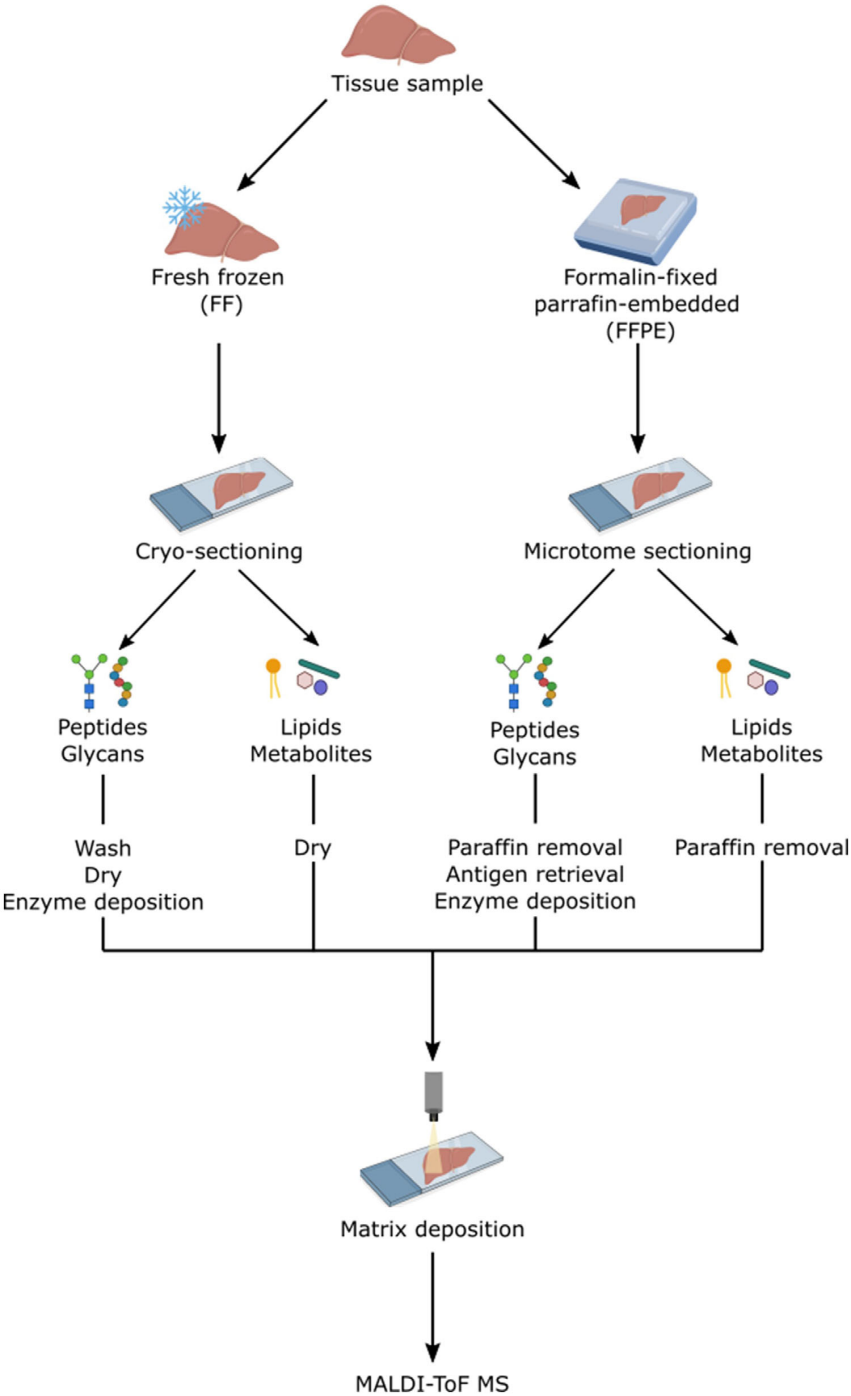
typical mass spectrometry imaging (MSI) tissue sample preparation workflow. Fresh‐frozen (FF) or formalin‐fixed paraffin‐embedded (FFPE) tissue is sectioned and mounted onto ITO slides. The tissue can be subjected to antigen retrieval (FFPE for peptides and glycans). A spray instrument is used to spray an enzyme, depending on the analyte of interest, directly onto the tissue section followed by spray application of matrix. Mass spectra are acquired across the tissue section and the data are analyzed using MSI software

The preparation of tissue sections to preserve the morphology and chemical information of analytes is one of the most crucial aspects of MSI.^12^ The sample preparation workflow typically involves (1) fixing, (2) embedding, (3) preparation (sectioning, mounting and washing), and (4) enzyme and matrix deposition. However, depending on the tissue‐type being analyzed, the sample preparation methods will vary widely (Figure 2). From a clinical perspective, application of MSI for the analysis of formalin‐fixed paraffin‐embedded (FFPE) tissue is imperative for the technique to be adopted as a tissue‐based diagnostic.^5^ The optimization and standardization of the sample preparation protocols is essential for production of high quality MSI data. This section will review some of the most recent aspects of the sample preparation workflow in MSI and its progression during the past 12 months, with an emphasis on MALDI‐MSI and FFPE tissue.

### 2.1 Tissue fixation

Tissue fixation is required after collection to halt enzymatic activity and reduce delocalization of analytes across the tissue. FFPE tissue has been a mainstay in pathology laboratories for decades, because of its near‐infinite shelf life compared to FF tissue. The fixation process leads to crosslinked proteins, which makes intact protein imaging difficult or near impossible.^13^ Additionally, the use of chemicals for fixation and those used for subsequent de‐paraffinization can lead analyte delocalization across the tissue.^14^ Recently, the use of different tissue fixatives was evaluated to determine whether they delocalize and suppress small metabolites of interest using gastric cancer tissue.^14^ There were no signs of analyte delocalization and that the detection of specific metabolite classes was dependent on the fixative used. This suggested that careful experimental design is required for tissue procurement depending on the type of analyte being monitored. Recently, an ethanol‐fixed paraffin‐embedded (EFPE) method was used on human hippocampal tissue sections from Alzheimer's disease patients.^13^ Here, the fixation of tissue with ethanol does not covalently cross‐link the proteins and this facilitated the monitoring of intact proteins and the spatial imaging of beta‐amyloid plaques.^13^

### 2.2 Tissue embedding

Traditional tissue embedding media that are used to assist with tissue sectioning and preserve intact tissue morphology for histology purposes are optimal cutting temperature medium (OCT) and hydrogels (eg, polyethylene glycol).^15^ However, not all embedding media are suitable for MSI as OCT causes ion suppression.^16^ Alternative embedding media have been used such as carboxymethyl cellulose (CMC),^17^, ^18^ gelatin,^19^ and poly N‐(hydroxypropyl)‐methacrlyamide (HPMA)^20^ that are compatible with downstream MSI. Recently, a universal embedding protocol (using a hydroxypropyl methylcellulose (HPMC) and polyvinylpyrrolidone (PVP) mix) was investigated that allowed sample analysis on a variety of MSI technologies including SIMS, desorption electrospray ionization (DESI) and MALDI, and was compatible with immunohistochemical (IHC) processes.^21^ Despite recent developments of MSI compatible embedding medium, their use for fragile plant tissues remains challenging. A novel poly‐l‐lysine (PLL)‐based tissue embedding method was developed for the analysis of plant tissues that reduced background signal and retained tissue topography.^22^ Additionally, PLL has the potential to be used for embedding other fragile tissues (e.g. bone).

The great availability of FFPE tissue samples have allowed the study of various clinical diseases and is routinely used for protein/ peptide profiling. However, their use for the analysis of lipids remains challenging as they are depleted by the paraffin‐embedding and washing steps currently used in FFPE sample preparations.^23^ Recent work has shown that the use of antigen‐retrieval (AR) steps on FFPE tissues enabled an increased number of lipid species to be identified.^24^ These studies highlight the potential applicability of spatial lipidomics from FFPE in clinical settings. The advancement of lipidomic MSI using FFPE tissue is dependent on improvements to sample preparation and a further understanding as to how these approaches affect lipid delocalisation and abundance.

### 2.3 Tissue preparation

Tissue thickness is an important aspect of MSI sample preparation as different tissue thicknesses can lead to variable data. The standardization of tissue thickness for different sample types (FF or FFPE) and MSI platforms is important for use of these technologies as a clinical diagnostic. A recent study evaluated the impact of tissue section thickness on FFPE tissue microarrays using MALDI‐Time‐of‐Flight MS (MALDI‐TOF MS).^25^ Here, it was shown that higher signal intensities were observed for 1 µm tissue sections compared to 5 µm tissue sections. They also report the difficulties in obtaining thin tissue sections (1 µm) for each core due to floating and rolling, and state that larger sample sizes are required to fully elucidate optimal tissue thickness. Further, they suggest that although 1 µm may provide the highest sensitivity, compatibility with IHC techniques may require thicker sections. This study highlights the importance of sample preparation standardization to enable the use of MSI as a potential tissue‐based diagnostic in clinical settings.

Preparation of tissue for subsequent MSI analysis requires optimized preparation per tissue type. Typically, coated (e.g. indium–tin oxide coated; ITO) glass slides are utilized for tissue mounting in MALDI and SIMS MSI. The addition of an adhesive substance to ITO glass slides prior to tissue mounting has been shown to be beneficial as it prevents tissue loss during wash steps.^26^ Recently, we reported that pre‐coating ITO slides with gelatin and chromium potassium sulfate dodecahydrate significantly improved the adherence of fragile hard FFPE human cartilage‐bone tissue sections.^27^ The mounting of FF tissues such as bone, skin, lung, and fat onto ITO slides is difficult. An optimized method to prevent tissue loss during this process using an adhesive cryofilm of 3C(16UF) and 4D(16UF) that produced similar conductivity to ITO slides enabled the analysis of difficult tissues.^28^, ^29^

Tissue sections contain a diverse set of analytes that vary in their abundance. Sample pre‐treatment (tissue washing) can aid in the removal of abundant species, salts, and other molecules.^30^, ^31^ A recent study washed FF and post formalin‐fixed tissue sections with ammonium formate prior to matrix application to increase the signal of gangliosides in both rat and human brain samples.^32^ Similarly, a group utilized acetone washing to significantly improve the detection of metabolites in FF rat brain and spleen tissue samples.^33^ These studies highlight the important of tissue washing procedures to enhance sensitivity and reduce ion suppression in MSI analysis. However, it is imperative to test the washing procedure to ensure no delocalization of analytes occurs.

### 2.4 Enzyme and matrix deposition

The use of MSI for routine clinical applications, specifically MALDI‐MSI, requires the implementation of consistent matrix deposition. Matrix heterogeneity and crystal size can lead to issues with sample reproducibility, quantitation, and spatial resolution. The implementation of robotic sprayers (e.g. ImagePrep by Bruker Daltonics, HTX TM‐Sprayer™ by HTX Technologies LLC) and sublimation systems has enabled more consistent and reproducible matrix deposition. A challenging aspect of metabolomic MALDI‐MSI workflows is the selection of an appropriate matrix as its ionization causes interfering ions in the low mass range. Matrices such as 1,5‐diaminonaphthalene (DAN)^34^ and 1,1′‐binaphthyl‐2,2′‐diamine (BNDM)^35^ have been successfully implemented for MALDI‐MSI analysis of metabolites. Matrix‐free approaches have been discussed as an alternative to organic matrices, however, their routine use in MSI settings is limited. Recently, the feasibility of gold nanoparticles (AuNPs) with high lateral resolution (5 µm) for the detection of neurotransmitters and amino acids in the brain was investigated using MALDI‐MSI.^36^ Additionally, the use of a porous alumina membrane (surface‐assisted laser desorption/ionization) was developed for the analysis of metabolites from FF tissue.^37^ The alumina membrane produced a matrix‐free spectrum that was of lower intensity compared to those used with organic matrices. However, this system could be adapted for use on dried samples by the development of an external solvent for extraction. The use of a polydimethylsiloxane (PDMS) stamp containing matrix within the wells was utilized to extracts analytes from a precisely defined area.^38^ The adsorbed analytes are concentrated at the well, with walls between compartments to preserve spatial information, as illustrated by the quantitation of peptides across a silicon wafer.

Lastly, the use of multiple enzymes for the analysis of a single tissue sample is advantageous where biological tissue is limited. The use of a serial enzyme digest strategy (chondroitinase, PNGaseF, elastase, and collagenase type III) was used to define the complex extracellular matrix (ECM), including post‐translational modifications (i.e. *N*‐glycans).^39^ Further, combining enzyme in situ digestion with nonionic surfactant pretreatment on FF brain tissue has recently shown to improve observable peptides, particularly above *m/z* 2500 Da. The use of additives with enzyme digestion provides an inexpensive alternative to improving MALDI‐MSI sensitivity, particularly for larger peptides.^40^

The development of novel matrices and matrix‐free applications have enabled the detection of a larger range of analytes and improved matrix crystallization (resolution). Similarly, the production of automatic sprayers has led to more reproducible enzyme and matrix deposition. Due to the costs associated with these automated sprayers, it has led to research groups developing their own systems.^41^, ^42^ The use of MSI for subcellular imaging (< 5 µm) requires sample preparation and matrix selection to be optimized for each sample type. Therefore, a major limitation that needs to be addressed prior to MSI use in routine clinical settings is the standardization of automated sprayers and matrices for specific analytes that balances resolution, specificity, and sensitivity. Future optimization of deposition to ensure shot‐to‐shot repeatability will greatly benefit quantitative MSI approaches.^43^

## 3 IONIZATION SOURCES

The most common MSI ionization techniques are SIMS, DESI, laser ablation‐inductively coupled plasma‐mass spectrometry (LA‐ICP‐MS), and MALDI. These ionization sources are coupled to a mass analyzer that enables the detection of molecules within a specified mass range. The ion source and mass analyzer determine the capabilities and limitations of each MSI technique and therefore, the applicability of each system needs to be optimized for their sensitivity and selectivity towards different molecules and tissue types. This section provides an overview of different MSI ionization sources and their developments in 2020.

With respect to spatial resolution, in comparison with other imaging modalities (e.g. confocal microscopy), MSI is limited. Advances in ionization sources have seen the achievable spatial resolution increase to allow cellular imaging, and Table 1 provides an overview of the different ionization sources and their respective advantages and limitations.

**TABLE 1:** Ionisation sources in MSI

Ionisation source	Spatial resolution	Application	Advantages	Limitations	References
nanoSIMS and SIMS (high vacuum)	0.05‐0.10 µm	Lipids, metabolites, small metabolites	Minimal sample preparation	Limited mass range, sensitivity	^128^
DESI	50‐200 µm	Lipids, metabolites	Ambient conditions, minimal sample preparation	Limited mass range, spatial resolution	^52^
nanoDESI (ambient)	10 µm	Lipids, metabolites, small peptides	Ambient conditions, minimal sample preparation	Technical adjustment of shear‐force probe and nano probe	^129^
LA‐ICP‐MS (Vacuum)	1‐100 µm	Elements	High sensitivity	Limited analytes, destructive technique	^130^
MALDI (ambient)	1.4‐3 µm	Proteins, peptides, lipids, sugars, metabolites and volatiles	Higher spatial resolution (compared to traditional MALDI)	Sample preparation, matrix interferences	^58^
MALDI (high vacuum)	10‐100 µm	Proteins, peptides, lipids, sugars, metabolites	Large mass range (0‐100000)	High vacuum, matrix interferences	^56^, ^57^, ^131^

The SIMS platform works by directing a focussed primary ion beam directly onto the tissue, to generate secondary ions.^44^ The primary advantage of SIMS ionization is the ability to obtain high spatial resolution (50‐250 nm) and unlike other MSI ionization techniques, SIMS allows the user to analyze the tissue directly without using matrices to facilitate transition into the gas phase.^45^ Traditionally, SIMS was limited to the analysis of elemental composition, however, the integration of gas cluster ions (Au_3_^+^ and Bi_3_^+^) has enabled the analysis of small biomolecules such as lipids.^46^ In practice, SIMS suffers from extensive ion fragmentation which results in poor sensitivity and limits the analysis to small analytes (*m/z* < 1000).^45^

Metal ions have long been implicated to play important biological roles.^47^ LA‐ICP‐MS is ideally suited to monitor the spatial distribution and intensity of elements across multiple tissue types in the context of pathological disease.^48^ Typically, a pulsed laser beam irradiates the tissue sample, generating an aerosol that is directed to the mass analyzer by a carrier gas.^48^ LA‐ICP‐MS has good spatial resolution (1‐100 µm) and offers excellent sensitivity (sub µg/g).^49^ However, this technique has long acquisition times and requires complementary MSI techniques to characterize metals in complexes (e.g. metalloproteins, metal‐binding proteins, and phosphoproteins).^50^

DESI is a technique performed at atmospheric pressure that is suited to the analysis of small molecules (<2000 Da) and does not require extensive sample preparation (fixation or matrix preparation).^51^ This technique utilizes electrosprayed liquid droplets that are directed onto the tissue surface. The ions are desorbed from the sample by electrostatic and pneumatic forces before being transferred to the mass analyzer by an atmospheric pressure ion‐transfer line.^51^ Despite DESI being a less destructive ionization technique and offering good sensitivity, it suffers from limited spatial resolution (200 µm) with respect to cellular imaging.^52^ More recently, a nano‐DESI technique was developed that was able to achieve 10 µm spatial resolution when imaging relatively flat tissues (brain and lung).^53^ This was achieved by adjusting the widths of the capillaries within the DESI probe and implementing a shear force probe to ensure droplets were within 1 µm of the tissue.^53^

MALDI is the most widely used MSI technique and can characterize many analytes such as proteins, peptides, glycans, metabolites, and lipids.^54^ Typically, tissue samples are cut at 1‐20 µm section thicknesses before being coated in an organic matrix. The most commonly used matrices for MALDI analyses are α‐cyano‐4‐hydroxycinnamic acid (CHCA), 2,5‐dihydroxybenzoic acid (DHB), and sinapinic acid (SA), with each preferentially ionizing a specific class of molecule. The matrix‐embedded analytes are continually ablated by a laser pulse, resulting in absorption of the laser energy by the matrix and the desorption/ionization of the sample and matrix molecules into a gaseous plume.^55^ The mass spectra at each point are correlated to a specific *x* and *y* coordinate to provide spatial localization of the molecules across the sample section. Traditional MALDI ionization sources have good resolution (10‐100 µm) and sensitivity, but recent improvements in laser technology have lowered this range.^56^, ^57^

**FIGURE 3:**
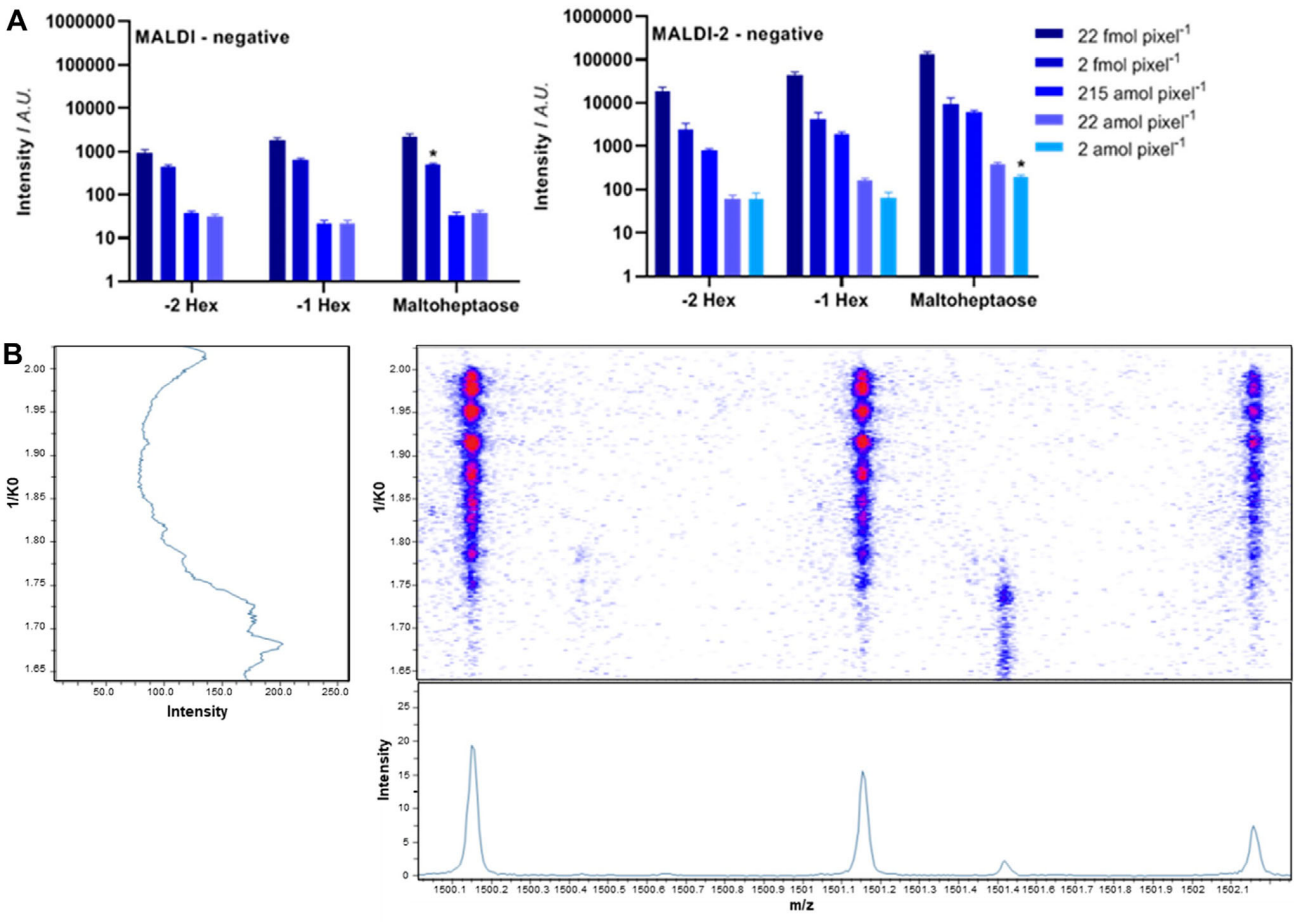
Improvements to MALDI‐MSI instrumentation in 2020. A) The use of MALDI‐2 post‐ionisation to improve oligosaccharide sensitivity. A dilution series of spraycoated maltoheptaose on glass slide was analyzed by MALDI‐2‐MS in negative ion mode (Left) and MALDI‐MS in negative ion mode (Right). The bars show the mean intensity of the performed experiments (n = 5). Asterisks (*) highlight the lower limit of detection (signal‐to‐noise ratio (SNR) ≥ 3), and error bars represent the SEM. Image adapted from Heijs et al.^63^ (https://pubs.acs.org/doi/10.1021/acs.analchem.0c02732) with copyright permission. Copyright (2020) ACS Publications. B) Separation of N‐glycan (m/z 1500) structural isobaric isomers from patient endometrial cancer (EC) tissue using a MALDI timsTOF flex. Patient EC tissue was prepared as described in Ref. 7

An atmospheric pressure MALDI (AP‐MALDI) source that could achieve a spatial resolution down to 1.4 µm,^58^ and another AP‐MALDI source configured with a 213 nm laser (compared to tradition sources that are 337 nm) that still maintained high resolution (3 µm) has enabled the use of alternative matrices, thus extending the capabilities of MALDI.^59^ The ability to obtain high spatial resolution while maintaining ionization efficiency is fundamental to the advancement of the MSI field. The resolution of MALDI instruments determines the ion yield and therefore, as the spatial resolution is improved, the number of ions generated becomes restricted (on average 1 in 1000 desorbed ions are ionized).^60^ To increase the detection of neutral ions and low abundance molecules on high resolution MALDI sources, new generation MSI platforms with MALDI‐2 post‐ionization (PI) have been reported.^54^ An additional orthogonal laser is fired into the gas plume, ionizing neutral matrix molecules that allows subsequent charge transfer to the analyte of interest.^61^ This results in more ionized analytes per laser pulse and an increase in sensitivity of up to three orders of magnitude. Recent studies have shown that the addition of MALDI‐2 PI allowed the analysis of labile compounds (e.g. lipids)^60^, ^62^ and post‐translational modifications (PTM; e.g. glycosylation).^63^ Heijs *et al* showed improved sensitivity of maltohepatose using the MALDI‐2 PI source of 3 and 2 orders of magnitude in negative and positive modes, respectively (Figure 3A).^63^ The ability to analyse the distribution of analytes is important as many pathological diseases are heterogeneous in nature.^64^ Although MALDI‐2 PI allows more analytes of interest to be detected after each laser pulse, this results also in an increase in mass spectrum complexity. As many analytes are isobaric in nature (eg, lipids and glycans) and only differ in their spatial conformation, it has led to the requirement of an additional separation technique to obtain increased sensitivity. The primary disadvantage associated with MALDI‐MSI is the complex sample preparation protocol that requires the extraction of analytes to the surface (eg, peptides and glycans) and use of a matrices that can affect surface homogeneity and interfere with analysis in the low mass range. Analyte extraction is also critical for quantitative MSI, with tissue composition known to affect extraction efficiency compounding variation due to adopted matrix preparation.^43^, ^65^

## 4 MASS ANALYZERS

MSI employs several MS configurations, including axial TOF MS,^66^ orthogonal TOF,^67^, ^68^ FT‐ICR,^69^, ^70^ Orbitrap,^67^, ^71^, ^72^ and QqQ^73^ MS with an ever‐increasing number of choices and capabilities.

For ESI/MALDI‐MSI, 2020 has seen the introduction of multiple configurations from vendors, including the ability to couple hardware from different suppliers.^74^ Spectroglyph LLC has developed an ESI/MALDI ion source that is compatible with many Orbitrap mass analyzers. The Spectroglyph source can be connected to any Thermo instrument with the ionmax/s‐lens source (e.g. Orbitrap Elite, QExactive, and so on, but not Fusion, Lumos, Exploris 480, etc). The source can be configured to allow for a MALDI‐2 laser. Much of the MALDI‐2 work has been done so far on Orbitrap instruments configured with a MALDI‐2 set up at the University of Münster (Germany),^54^ Maastricht University,^75^ and at the University of Wollongong (Australia).^75^ In June 2020, an optimized infrared MALDESI (IR‐MALDESI), RastirX, was developed to enable the analysis of samples with arbitrary patterns and coupled to a Q Exactive HF‐X.^76^ In June 2019, Bruker Daltonics released the ESI trapped ion mobility mass spectrometer (ESI‐tims‐QTOF‐MS), and the timsTOF fleX with a dual MALDI/ESI ion source that features an additional trapped ion mobility spectrometry (TIMS) capacity. In June 2020, Bruker Daltonics subsequently released a MALDI‐2 PI source for the ESI/MALDI timsTOF fleX MS. It has been shown that the implementation of TIMS in conjunction with MALDI‐2 PI and TOF capabilities led to a substantial increase in the number of identified features for imaging of lipid distribution on rat brain tissue^62^ and greater discrimination of metabolic analytes in human kidney tissue.^77^ The MALDI timsTOF flex MS can also being used to separate structural isobaric isomers of *N*‐glycans directly from tissue as shown in Figure 3B.

The ability of DESI systems to analyze tissue samples at higher spatial resolutions (<20 µm) has permitted the characterization of lipids in glioblastoma^78^ and perfluoroalkyl substances in plant tissues.^79^ Recently, Waters Corporation released the cyclic ion mobility (IM) mass analyzer that can be combined with a DESI XS ionization source. The cyclic IM mass analyzer offers multiple fragmentation options, including collision‐induce dissociation (CID), electron‐capture dissociation (ECD), and surface‐induced dissociation (SID). Additionally, this system is the first to perform IM fragmentation analysis (IMS^n^) whereby selected segments of the structure can be isolated and stored, while the unwanted ions are ejected. The stored ions are re‐injected and can undergo additional IMS^n^ cycles to increase the structural characterization of the analytes.

Typically, a tissue sample for MSI analysis is observed as a whole image using an external scanner. Shimadzu have released a new platform, the iMScope™ QT, which is equipped with an optical microscope in conjunction with MSI and Q‐TOF MS analysis. The sample tissue is placed onto a glass slide, inserted into the instrument and by using the in‐built optical microscope, microscopic regions of interest can be selected and analyzed as part of an integrated workflow. A recent study utilized this workflow to assess the distribution of glycoalkaloids in potato tubers and how they altered with respect to storage times.^80^

New developments in MSI instrumentation have revolutionized the field, with the ability to achieve high spatial resolution, increased sensitivity, and quantitative capabilities. The use of higher resolution instruments has been shown to lead to a loss in sensitivity due to the smaller number of ions generated. However, the advent of PI sources (e.g. MALDI‐2) shows promise in circumventing this issue. An emphasis will need to be placed on optimal sample preparation to preserve the structure and localization of analytes within sample tissue. For high‐resolution instruments, continued improvements in data acquisition time and data processing times are required to meet high‐throughput capabilities for routine clinical use.

## 5 DATA ANALYSIS

Data analysis is an integral aspect of any MS workflow. Irrespective of the ionization technique employed, all MSI analyses result in data acquisition from a tissue across the *x* and *y* dimensions. The extraction of meaningful information from MSI data poses a challenge due to the high dimensionality (spatial information) that needs to be retained and the large size of data files. As most MSI analyses adopt a non‐targeted approach, data analysis becomes more complex and the extraction of consistent and high‐quality information is required.

**FIGURE 4:**
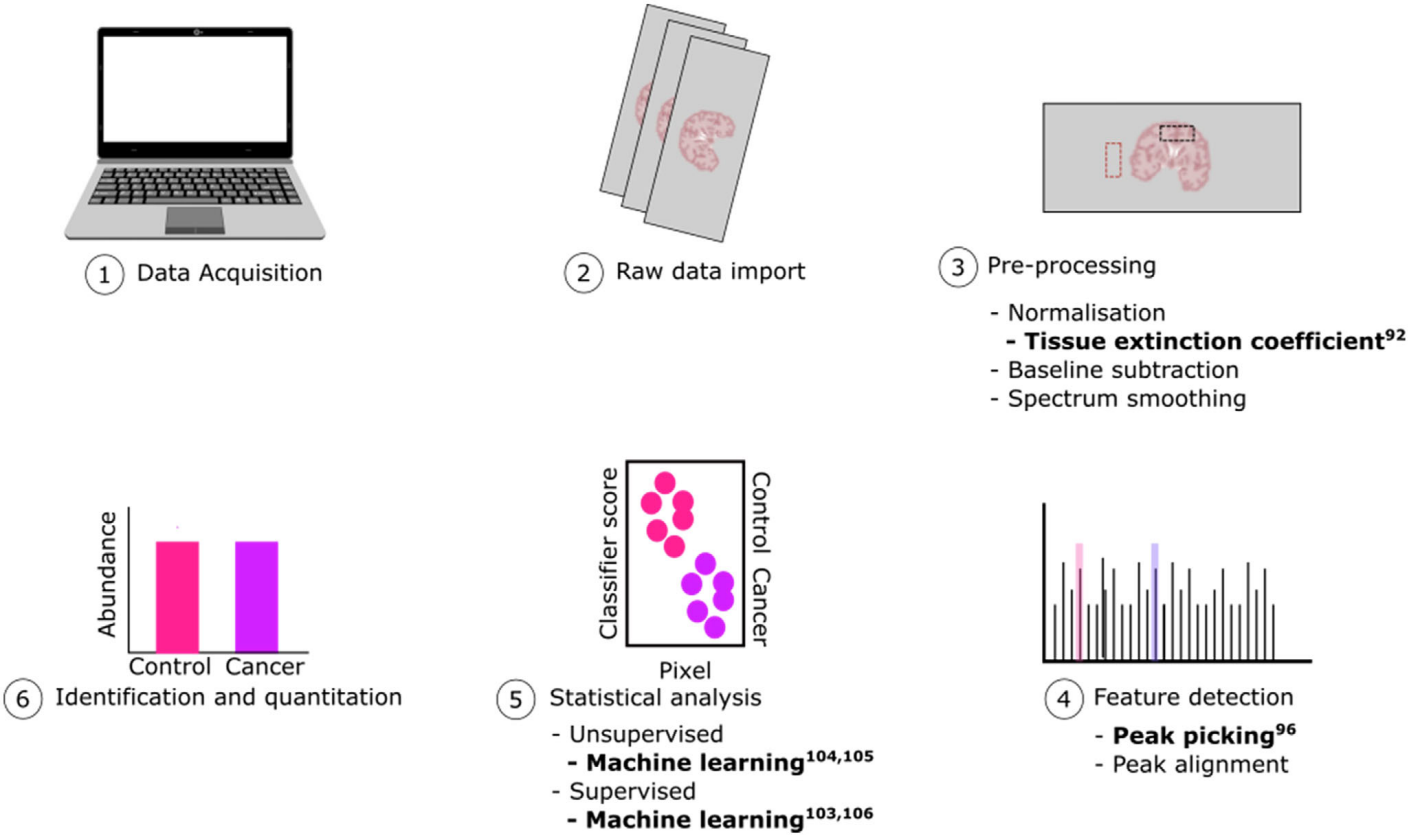
A typical mass spectrometry imaging (MSI) data analysis pipeline. Data from mass spectra acquired from a tissue sample are imported and subsequently pre‐processed prior to feature detection, statistical analysis, and identification and quantitation

Currently, there are a range of vendor‐dependant software's for MSI data analysis, including flexImaging and SCiLS lab (Bruker Daltonics), ImageQuest (ThermoFisher Scientific), High Definition Imaging (Waters), and Imaging MS Solution (Shimadzu). Many of these vendor software packages offer the capability to export datasets in the common data format imzML,^81^ which is designed to contain a complete description of the imaging experiments and allow efficient data storage. These formats allowed the development of open‐source environments such as R (MALDIquant^82^; Cardinal^83^; rMSIproc^84^), MATLAB (MSireader^85^), and Datacube Explorer.^86^ Further, the use of imzML formats allows MSI researchers to directly access published data sets. The data analysis approach can vary depending on the type of MSI experiment as well as the analytes measured. Nonetheless, a typical data analysis pipeline involves (1) raw data import; (2) pre‐processing, including normalization, baseline subtraction, and spectral smoothing; (3) feature detection, including peak picking and peak alignment; (4) statistical analyses, and this can be combined with; (5) identification and quantitation (Figure 4).^87^

An important aspect of MSI pre‐processing is spectra normalization. Normalization processes are employed to scale each spectrum to a certain factor to enable equivalent comparisons of mass intensities from different spectra.^87^ In MALDI‐MSI, normalization is utilized to reduce the influence of heterogeneous matrix crystallization and ion source degradation. The most common approach for spectra normalization is to divide each mass intensity by the sum of all intensities across the spectrum, otherwise known as the total ion count (TIC). More recently, the use of a “tissue extinction coefficient” (TEC) can be used to evaluate ion suppression effects across different regions of interest (ROI) and tissue types.^88^, ^89^ Here, a standard is deposited with the matrix onto the control tissue and its mean intensity is compared to a reference. This allowed quantitation of the antipsychotic drug olanzapine in mouse tissue, with comparable results to liquid‐chromatography MS/MS (LC‐MS/MS).^89^ Subsequently, standard methods for pre‐processing (smoothing and baseline correction) can be used for imaging data.^90^

After pre‐processing, peak picking algorithms are employed to detect pronounced peaks from the background noise. In MALDI‐MSI the ability to accurately differentiate between sample peaks and noise becomes inherently more difficult as low mass regions have increased noise levels. The peak picking algorithm employed is crucial to successful data analysis as subsequent analysis (e.g. component analysis and spatial segmentation) rely on accurate peak identification. Traditional algorithms for peak picking such as the average‐spectrum approach suffers in its ability to detect low‐intensity peaks and do not fully account for spatial information.^91^ The detection of peaks using spatially structured *m/z*‐images instead of spectra‐wise methods was explored by Alexandrov and Bartels.^91^ However, this type of peak picking is hindered in its ability to differentiate peaks in noisier QTOF data sets.^92^ More recently, the combination of spatial structure and traditional peak picking algorithms that detected peaks and their spatial distribution was implemented on MALDI‐TOF data.^93^

Statistical analyses can be divided into supervised and unsupervised approaches. After spectral pre‐processing (e.g. peak picking, peak alignment, normalization, etc), supervised analyses can be performed by providing the specification of at least two groups (e.g. tumor vs control regions), whilst unsupervised clustering requires no prior knowledge.^87^ The two main unsupervised visualization approaches typically adopted for untargeted MSI data analysis are component analysis and spatial segmentation. Component analysis encompasses principle component analysis,^94^ probabilistic latent semantic analysis,^95^ non‐negative matrix factorization,^96^ and maximum auto‐correlation factorization.^97^ The most common unsupervised visualization approach is spatial segmentation, where spectra are grouped based on their similarity and presented by an image that highlights regions of similarity.^87^ After segmentation, correlation algorithms are applied to detect *m/z* values that are co‐localized to a specific region. Recently, the use of machine learning (ML), specifically “deep learning,” has flourished in its ability to perform pattern recognition.^98^ Common ML approaches include neural networks (NN), linear vector quantization, self‐organizing map (SOM), support vector machine, and random forest classifiers.^99^ As MSI data is becoming increasingly complex, the use of NN deep learning algorithms has been successfully applied in various MSI experiments such as pulmonary arterial hypertension mechanisms,^100^ mouse tumor tissue,^101^ and polymer surface chemistries.^102^ To date, the majority of ML algorithms have been focused on the generation of 2D spectral images. The advent of the clinically relevant 3D‐biological models such as spheroids and organoids has highlighted the importance to investigate the interactions of cell clusters and their heterogeneity through topological mapping.^103^, ^104^ Gardner *et al*. demonstrated the use of SOM‐relational perspective mapping algorithms for 3D surface characterization and outlined the potential for this approach to be utilized for cell images.^105^

Data analysis is a major bottleneck for processing of data acquired using high resolution MSI instruments, due to increased data sizes and spectral complexity, limiting the current imzML format.^106^ Additionally, data analysis software that integrates MSI data with other fields (e.g. transcriptomics) is required for more comprehensive exploration of biological pathways in disease. While MSI is a technique currently adopted in industry settings, such as for *in vivo* pharmaceutical monitoring,^11^ focussed efforts in improving processing capabilities are needed to see implementation of MSI as a routine diagnostic approach. Improving analysis pipelines for clinical diagnostics will require ground truth data and access to high‐quality datasets for proper assessment of developed approaches, which is a major limitation at present.^106^

## 6 CELLULAR AND SUBCELLULAR IMAGING

Most mammalian cells are between 5 and 100 µm in diameter and therefore single cell MSI is generally performed at a spatial resolution of ≤10‐µm. The increased time for annotation and co‐registering of every single cell for high‐spatial resolution MALDI‐MSI has recently been reduced by using an ML approach, which semi‐automatically annotates single‐cells.^107^ Recent developments, that utilize 3D cell cultures to depict disease microenvironments more accurately have led to the improvement of MSI instrumentation such that it can be applied in clinical settings.^108^

In addition, MALDI‐MSI lipidomic analysis has been used to monitor lipids in mammalian retinal ganglion cells in situ and in vitro, by printing whole cells from primary cultures onto glass slides,^109^ as well as imaging lipids on a cellular and subcellular level and to initiate a lipid atlas of human retina and supporting tissues.^110^

The use of complementary techniques has aided the progression of MSI for single‐cell analysis. Recently, single‐cell metabolomics has been achieved by the combinational use of fiber‐based laser ablation electrospray ionization (f‐LAESI) with 21T FT‐ICR mass spectrometry (21T FTICR‐MS) in plant tissue with 47 known and 11 unknown compounds being monitored.^111^ The combination of peroxidase labeling of lysosomes and the metabolic labeling of proteins allows for multi‐isotope imaging mass spectrometry (MIMS) with a lateral resolution <50 nm.^112^, ^113^ The uptake of this technique in cell biology remains limited, because it relies on the availability of specific labeling. Similarly, the absolute quantification of organelle‐associated pro‐drug is feasible, but requires the isotopically labeled standard.^114^

TOF‐SIMS has been used to quantify drugs and metabolites on a subcellular level using nanoSIMS imaging and isotopically labeled pro‐drug.^114^ TOF‐SIMS has also been the method of choice to investigate the inter‐and intratumoral heterogeneity of glioblastoma,^115^ providing further inside into the distribution of proteins and metabolites with 800 nm spatial resolution.

To overcome the limitations of TOF‐SIMS to image biomacromolecules such as proteins, a genetic incorporation of fluorine‐containing unnatural amino acids as a chemical tag into the proteins was developed. This not only allows the visualization of proteins, but also the interactions between proteins and drugs.^116^ In a landmark study by Pareek and colleagues, subcellular imaging directly visualized *de novo* purine synthesis catalyzed by a multienzyme complex called the purinosome.^117^ They used metabolomics and high‐resolution gas cluster ion beam SIMS (GCIB‐SIMS) with a focal diameter of 1 µm, which makes the detection of the multiprotein with an estimated size between 0.2 and 0.9 µm in diameter feasible. Their data support the hypothesis that metabolomics pathways are biosynthetic “hotspots” within the cell.

## 7 CONCLUSION AND SUMMARY

2020 has been an exciting year for MSI, with significant progress being made across the MSI workflow. These developments have been driven by the development of coupling of high‐resolution mass analyzers with MSI ionization sources in conjunction with an improved data processing pipeline.

SIMS‐TOF offers, by far, the highest MSI resolution (<100 nm for inorganic species), however, its applicability for a wide range of analytes remains limited. The commercially available, MALDI timsTOF fleX, can typically achieve spatial resolutions of up to 10 µm at ∼20 pixels/s for a wide range of analytes. The iMScope™ QT can achieve spatial resolutions of 5 µm, however, has long acquisition times, whilst the DESI cyclic IMS can achieve spatial resolution of 20‐50 µm at a 50 Hz acquisition rate.

Sensitivity is a key issue associated with high‐resolution MSI instruments as the number of available ions is reduced. Advancements in ionization options with mass analyzers in the past 2 years have shown improvements in capability, yet continued development is required, particularly for targeted quantitation of analytes across tissue. This may be in the form of new generation ionization sources coupled to analyzers not currently available, such as MALDI/DESI coupled triple quadrupole (QqQ) MS, however, quantitative MSI of metabolites on existing MS platforms has already been shown to be comparable with conventional LC‐MS approaches.^89^, ^118^, ^119^ The implementation of PI sources (MALDI‐2 laser source from Bruker Daltonics and the MALDI‐2 capable source from Spectroglyph LLC) has also helped circumvent the issues associated with poor sensitivity on high‐resolution MSI instrumentation. The recent progress of research groups to retrofit MSI ionization sources with high‐resolution mass spectrometers results in increased analysis times. This may limit these applications use for routine applications. However, advances in laser optics have seen increases in scan speeds, with current state‐of‐the‐art MALDI systems achieving a scan speed of 20 pixels/s compared to earlier commercial systems that typically achieved scan speeds of 1 pixel/s.

The increased complexity of MSI data sets has led to the demand for software that provides advanced modeling for reliable statistical output. Additionally, the ability to correlate IHC data with MSI datasets is essential. The advent of imzML has led to the development of open‐source packages, more specifically, ML approaches that can be used with complex MSI data, but limited options with respect to quantitative MSI.^120^, ^121^ However, development of user‐friendly interfaces, allowing users with little MS‐expertise to access and analyze data, in a robust and validated workflow is required to see adoption of these methodologies in routine settings.

Lastly, continued development of standard operating procedures will facilitate the movement of MSI technologies for use in targeted quantitation and clinical applications, with QC requirements including sample preparation,^25^, ^122^ data acquisition,^123^ processing,^124^ reporting,^125^, ^126^ and for adopting quantitative MSI^127^ already under investigation. The routine use of PI sources (e.g. MALDI‐2 laser) could overcome limited sensitivity for some molecular classes and make them more accessible for diagnostic applications. The speed of the development of instruments with higher spatial and mass resolution have been astonishing and predict a bright future for MSI and its application.

## CONFLICT OF INTEREST

The authors declare no conflict of interest.
